# Spatial and temporal distribution of dengue in Brazil, 1990 - 2017

**DOI:** 10.1371/journal.pone.0228346

**Published:** 2020-02-13

**Authors:** Denise Catarina Andrioli, Maria Assunta Busato, Junir Antonio Lutinski

**Affiliations:** Postgraduate Program in Health Sciences, Community University of the Region of Chapecó (Unochapecó), Chapecó, Santa Catarina, Brazil; University of California, Davis, UNITED STATES

## Abstract

Dengue is a viral disease caused by an arbovirus of the genus *Flavivirus* transmitted in Brazil by the mosquito *Aedes aegypti* (Linnaeus, 1762). Simultaneous circulation of the four viral serotypes (DENV1, 2, 3 and 4) has been occurring since 2010 and determines a scenario of hyperendemicity of the disease in the country. This study aimed to describe the epidemiological situation of dengue in Brazil in the last three decades. This is a descriptive, observational study that used data of dengue notifications of the National Surveillance System from 1990 to 2017, available in the Epidemiological Bulletins and publications of the Ministry of Health. Dengue incidence increased in all Brazilian regions and the interepidemic periods are distinct in the different regions. The greatest epidemics was recorded in 2015 (1,688,688 cases), with an incidence of 826.0 cases per 100,000 inhabitants, which illustrates the occurrence of dengue in the last decade with increasingly higher epidemic peaks and shortening of the interepidemic periods. The incidence and mortality indices point to the need to improve the organization of response to dengue epidemics. This study provides information on the epidemiology of dengue in the country and can be used in the formulation of public health policies to reduce the impacts of viral transmission.

## Introduction

Dengue is the most common viral disease in the world transmitted by mosquitoes [1]. It is a Neglected Tropical Disease—NTD [2] that presents rapid worldwide dissemination, with a pandemic profile and represents one of the biggest problems of global public health [3]. Its incidence has been increasing in the last decades, expanding geographically to subtropical regions. Approximately 3.9 billion people live in more than 100 endemic countries at risk of dengue infection [4].

Defined as a feverish, acute and non-contagious disease, dengue is one of the most important arboviruses affecting humans. This is due to its high incidence and potential for dissemination. It is transmitted by infected *Aedes aegypti* (Linnaeus, 1762) and *Aedes albopictus* (Skuse, 1894), with *Aedes aegypti* being the urban vector responsible for transmission in Brazil [5].

The infection may be asymptomatic or symptomatic. When symptomatic, it ranges from milder forms to severe conditions, and may lead to death. The vector is cosmopolitan and occurs in tropical and subtropical countries where climatic and environmental conditions favor its dispersal and development. In addition, factors such as disordered city growth associated with poor sanitation also contribute to the spread of the vector. In Brazil, dengue is endemic and epidemic, with simultaneous circulation of the four viral serotypes already described: DENV1, DENV2, DENV3 and DENV4. In recent years, dengue has been spread in Brazil [6].

Several studies have demonstrated the economic and social impact on populations of endemic dengue areas [7–10], including economic impact in regions where the economy is based on tourism [11]. An estimate by Bhatt et al. [1] indicates that dengue is responsible for about 390 million infections per year, of which 96 million are apparent cases (clinically manifest). A growing incidence and geographic expansion of dengue outbreaks and epidemics in associated with the lack of effective vector control, lifestyle changes, population growth, urbanization, globalization and international human mobility [12,13]. Among these factors, urbanization probably influenced the amplification of dengue within countries, and international mobility had the greatest impact on the spread between countries [14].

The global growth of dengue cases and its emergence is related to the climatic, environmental, demographic and social changes of the last decades, including the population growth, the movement of people for trade, tourism or forced by natural disasters, political and economic factors, civil wars, disorganized urbanization, fragilities in public health and in the vector control program [7, 15].

The increasing number of severe cases and deaths due to dengue in the last two decades, the introduction and simultaneous circulation of chikungunya and Zika virus, whose symptoms are similar to those of dengue fever [16], pose additional challenges to the current epidemiological scenario of Brazil. Dengue control actions and knowledge on its propagation contribute to the prevention of these other arboviruses [17]. In addition, other viruses transmitted by the *A*. *aegypti* mosquito, already circulating in other Latin American countries, such as the Mayaro virus, add complexity to the definition of public policies for prevention and control [18].

Due to the magnitude of the epidemics, the presence of the four dengue serotypes in the country and the increasing probability of occurrence of severe cases of the disease, it is necessary to organize and plan appropriate actions aimed at vector control and disease prevention. The investigation of the epidemiological profile of dengue is important so that its magnitude is known, for the definition of public policies and the timely allocation of resources in the actions of control and prevention. On the other hand, it is also important and necessary to make a constant evaluation of the data generated by the epidemiological surveillance of the country. In this perspective, Bhatt et al. [1] reinforce that success in control requires strength of evidence in which control planning decisions are based.

It is considered important to use information to assist in the evaluation of the health situation for decision making, in order to direct intersectoral, educational and health education actions [19]. Observational studies in epidemiology have the potential to influence clinical practice, as they provide evidence and allow inferring spatial and temporal variations, projecting future scenarios. In this context, this study aimed to describe the epidemiological situation of dengue in Brazil in the last three decades.

## Method

### Context

The study area covered Brazil, the largest country in Latin America, with a geographical area of 8,510,820 Km^2^, in South America. The country is composed of 27 federative units, 26 states and a federal district distributed in five geographic regions: North, Northeast, South, Southeast and Center-West. Its population in the last census in 2010 was 190,755,799 inhabitants. The population estimate for the year 2018 was 208,494,900 inhabitants [20].

The Brazilian population density is 24.9 inhabitants per km^2^, with 58% of the population [21]. The climate in the country is tropical (most striking in the northeast, southeast and center-west), equatorial (northern) and temperate (southern). The temperature, humidity and rainfall vary from region to region and, in some regions, according to the seasons of the year [21].

### Study characterization

This is a descriptive observational epidemiological study that used dengue notification data from the National Surveillance System, considering the entire historical series from January 1990 to December 2017, available in the Epidemiological Bulletins and publications of the Brazilian Ministry of Health. Population data used to calculate incidence were obtained from the website of the Brazilian Institute of Geography and Statistics (*Instituto Brasileiro de Geografia e Estatística–IBGE*) [21].

### Statistical analysis

Data were tabulated in a database and descriptive statistics of frequency were used to describe the historical series of incidence and deaths from dengue in the period. In order to calculate the incidence rate, the number of reported cases was divided by the estimated population for each corresponding year. The result was relativized by groups of 100 thousand inhabitants.

A non-metric Multidimensional Scaling (NMDS) was applied to test for differences in the frequency and annual distribution of dengue between states of the federation. The matrix of the notifications was previously transformed into Log (x+1), Bray-Curtis was used as the association index and the analysis was performed in the statistical software Primer 6.1.9. [22].

The association of the annual notifications with the five Brazilian geographic macro-regions was tested based on a Principal Components Analysis (PCA). Data were previously transformed into Log (x+1) and analyzed with the aid of the software Past [23].

### Ethical aspects

This study did not require approval by the Research Ethics Committee, since public access databases were used, without identification of the cases.

## Results

In the period studied (1990 to 2017), the country has faced several dengue epidemics, characterized by a cyclical pattern, with more than 12 million reported cases. Of these, approximately nine million occurred in the last decade (2008 to 2017). The largest epidemics faced by Brazil were recorded in 2015 (1,688,688 cases), with an incidence of 826.0 cases per 100,000 inhabitants, followed by 2013 (1,452,489 cases) and 2016 (1,483,623 cases), with incidence of 722.5 and 719.9 cases per 100 thousand inhabitants, respectively (Table 1).

**Table 1:** Number of probable cases and incidence of dengue (/100 thousand inhabitants), Brazil, 1990 to 2017.

Year	Reported cases (n)	Incidence Rate (/100,000 inhab.)
1990	40279	27.4
1991	104399	71.1
1992	1696	1.1
1993	7374	4.9
1994	56691	36.9
1995	137308	88.1
1996	183762	117.0
1997	249239	156.1
1998	507715	313.8
1999	74670	45.5
2000	135228	79.6
2001	385783	223.8
2002	696472	398.8
2003	274975	155.5
2004	70174	38.6
2005	147039	79.8
2006	258680	138.5
2007	496923	270.1
2008	632680	333.7
2009	406269	212.2
2010	1011548	530.3
2011	764032	397.1
2012	589591	304.0
2013	1452489	722.5
2014	589107	290.5
2015	1688688	826.0
2016	1483623	719.9
2017	251711	121.2

**Fig 1:**
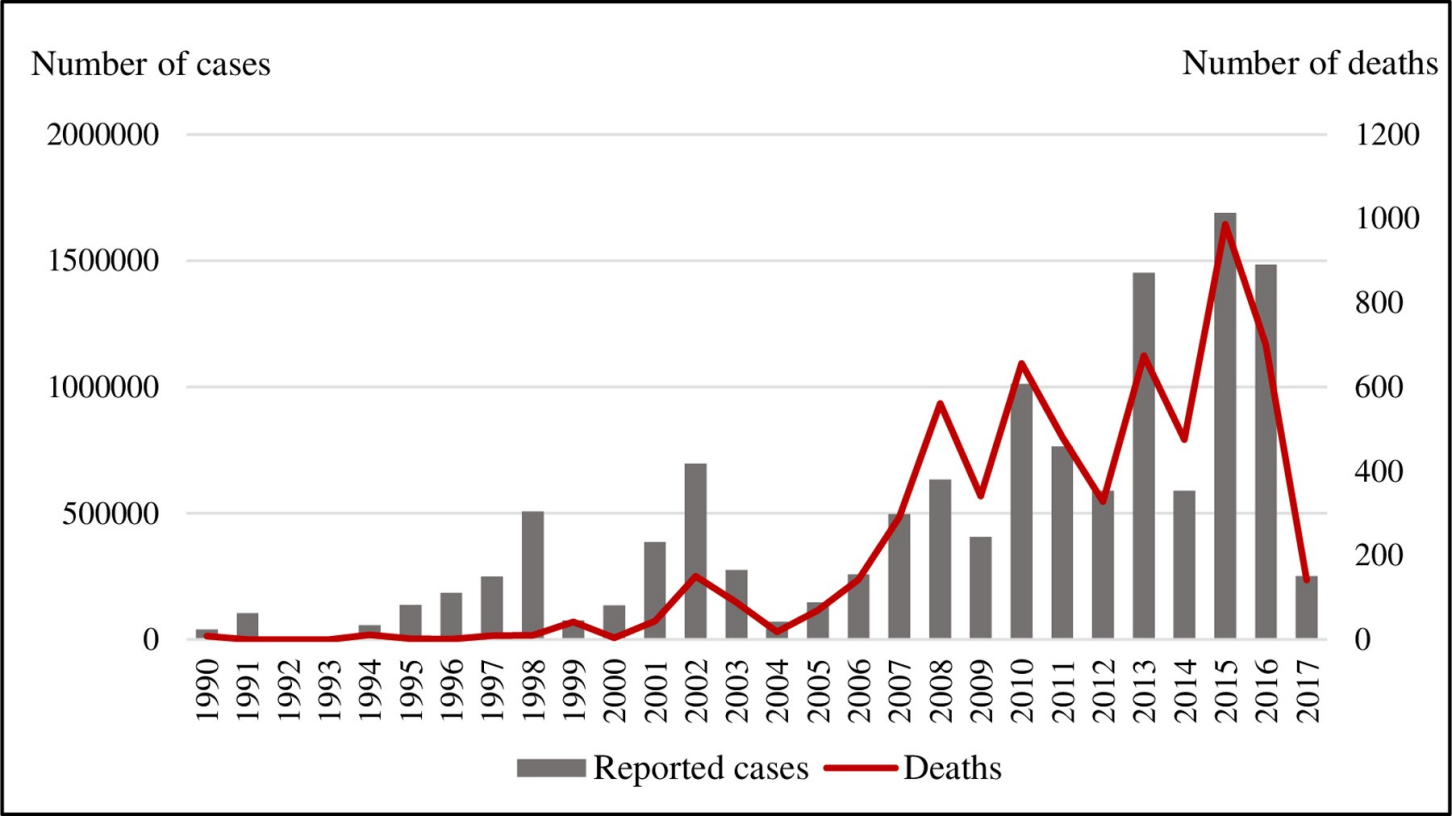
Reported cases of dengue and deaths in Brazil, 1990–2017.

Concurrently with the increase in the number of cases, there was an increase in the number of deaths, configuring a worsening tendency (Fig 1). The most significant increase in deaths was observed since 2008, especially 2015, when approximately 1,000 deaths were recorded, of which the state of São Paulo accounted for more than 50% deaths from dengue in that year. It is noteworthy that 2008, 2009 and 2014 were not the years with the highest number of cases, however they were the ones with the highest number of deaths compared to the reported cases. The lethality rate for dengue in the period ranged from 0.00 to 0.09.

**Fig 2:**
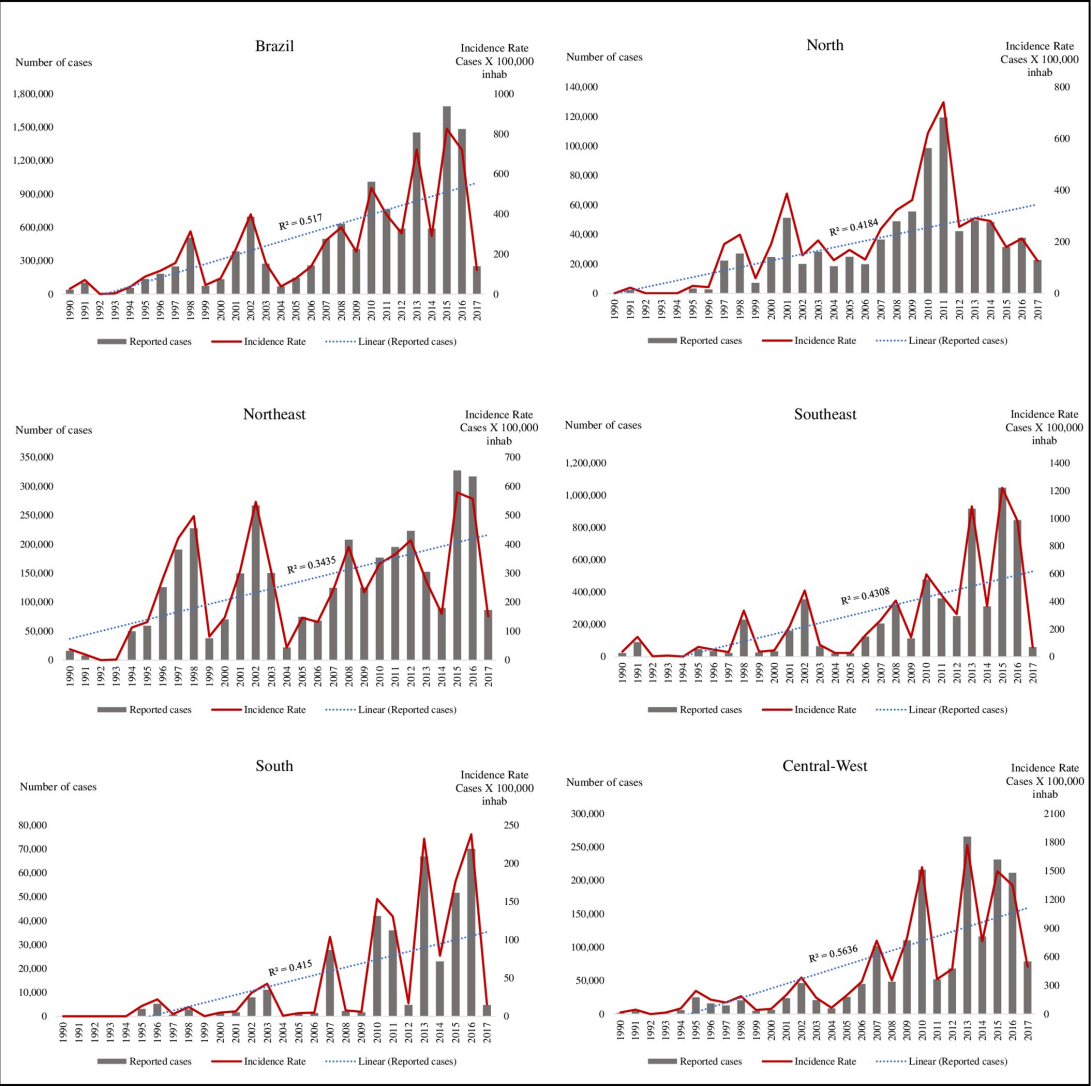
Annual incidence and cases of dengue by region of the country. Brazil, 1990–2017.

The region of the country with the highest number of reported cases of dengue fever was the Southeast, with 49% of the cases in the period, followed by the Northeast (28%), Center-West (14%), North (7%) and South (3%). However, the highest incidence was found in the Center-West, with an average rate of 444.8 cases/100,000 inhabitants, followed by the Southeast (272.7), Northeast (246.7), North (198.6) and South (46.7) (Fig 2).

The Southeast region, the most populous in the country and most affected by dengue, has a major influence on national dynamics. The interepidemic periods of this region have been shortened, resembling patterns of the country (Fig 2). In other regions, the interval of 3 to 4 years has remained.

**Fig 3:**
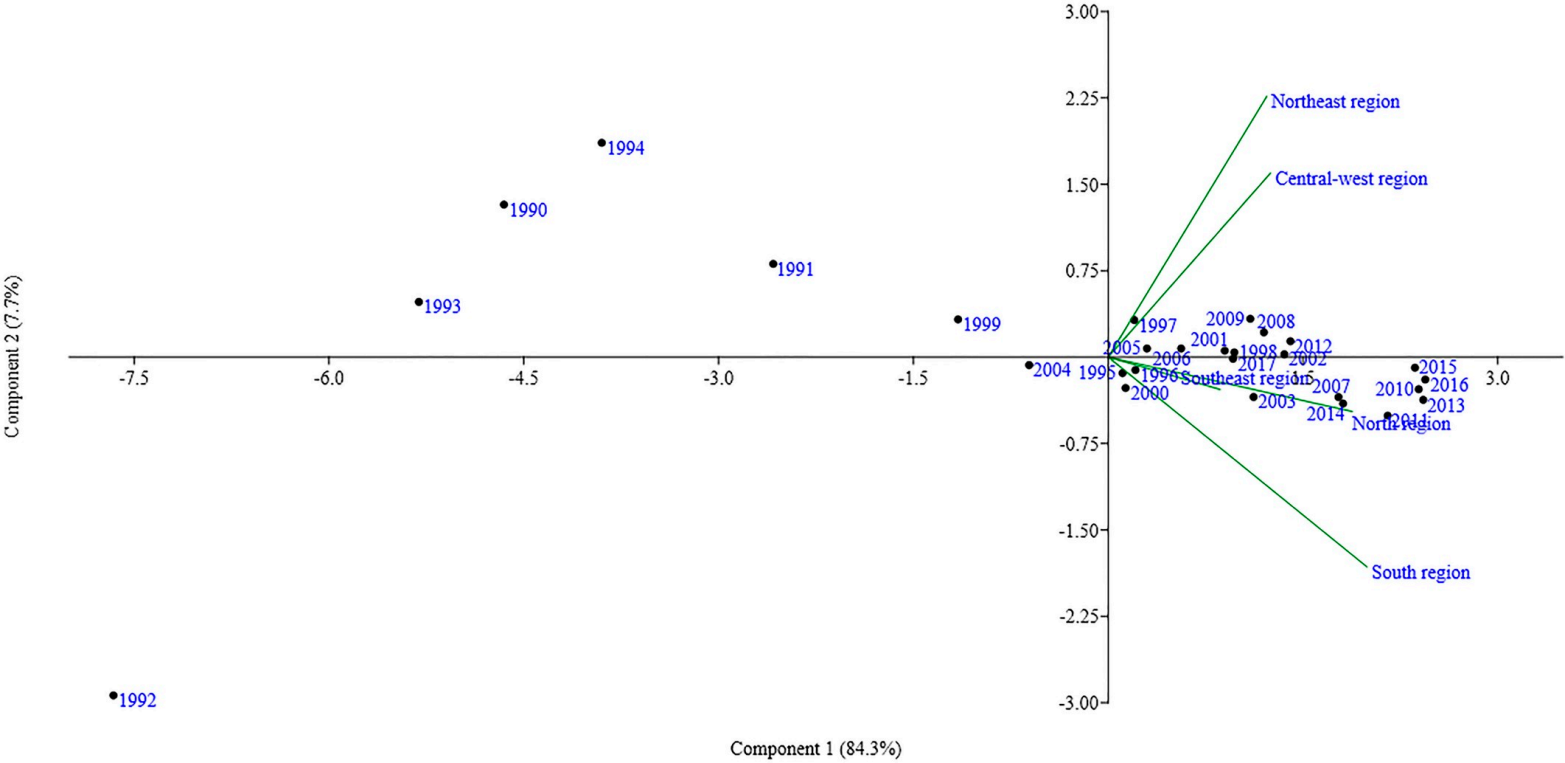
Association by the Principal Components Analysis (PCA) between annual dengue notifications and geographic macro-regions. Brazil, 1990–2017.

In all, 92% of the annual and regional variation of annual dengue notifications in Brazil were explained by PCA. The Center-West and Northeast regions showed similarity regarding the frequency of annual notifications. The North, Southeast and South regions had different profiles regarding notifications. The years 1990, 1991, 1992, 1993, 1994, 1999 and 2004 presented cases of dengue more generally. The notifications made in the other years showed associations with the Center-West and Northeast regions or with the North and Southeast regions. The notifications made in the South region showed no association with a specific year (Fig 3).

**Fig 4:**
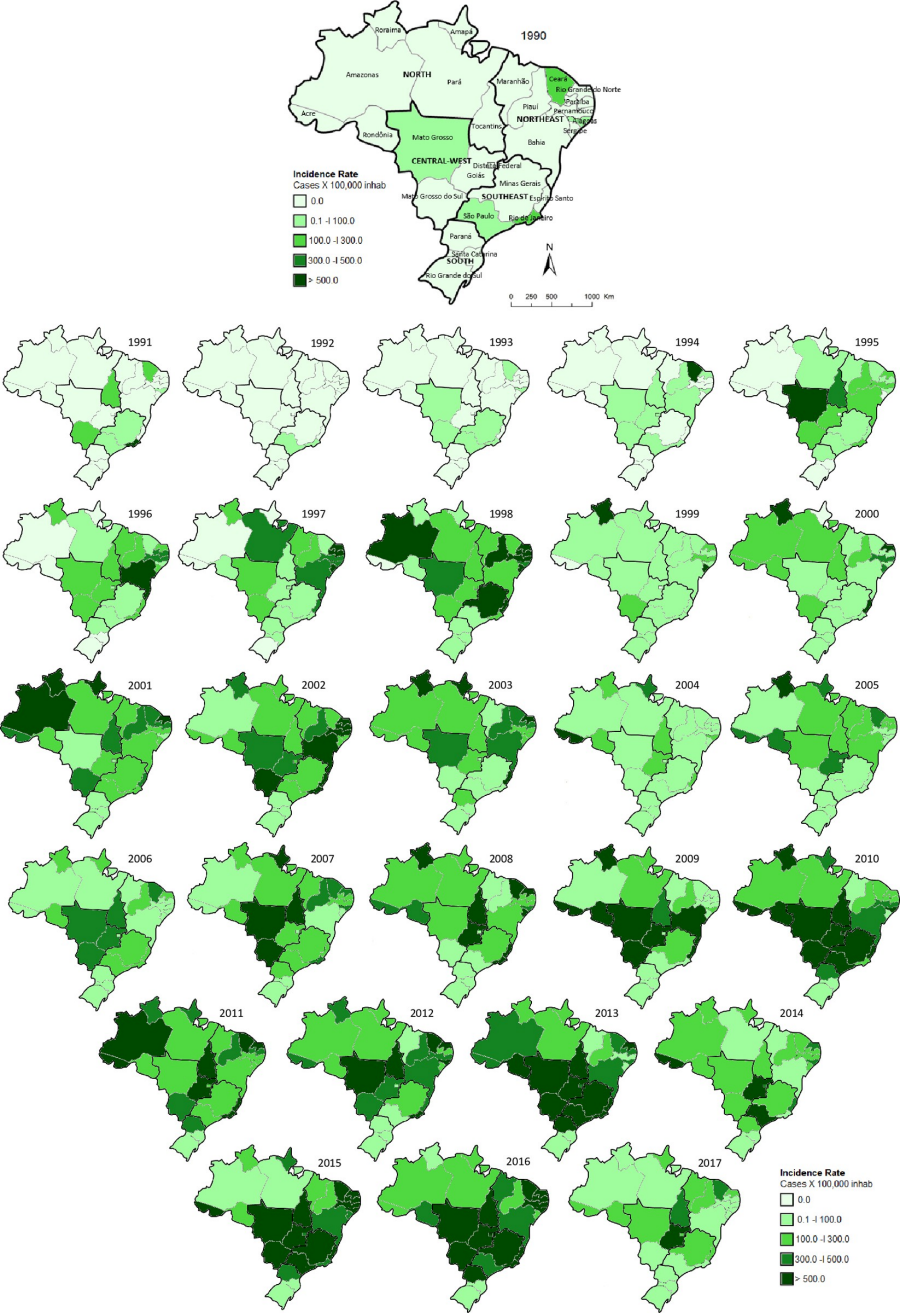
Spatial and temporal distribution of the incidence of dengue by state. Brazil, 1990–2017. Choropleth map produced using Paint of Microsoft Windows 10 version 1803 (© 2018 Microsoft Corporation). Source of Base Map: Brazilian Institute of Geography and Statistics (IBGE in Portuguese; https://portaldemapas.ibge.gov.br).

It is observed an dissemination and an increase in the incidence of dengue in the units of the Brazilian federation, during the evaluated period (Fig 4).

**Fig 5:**
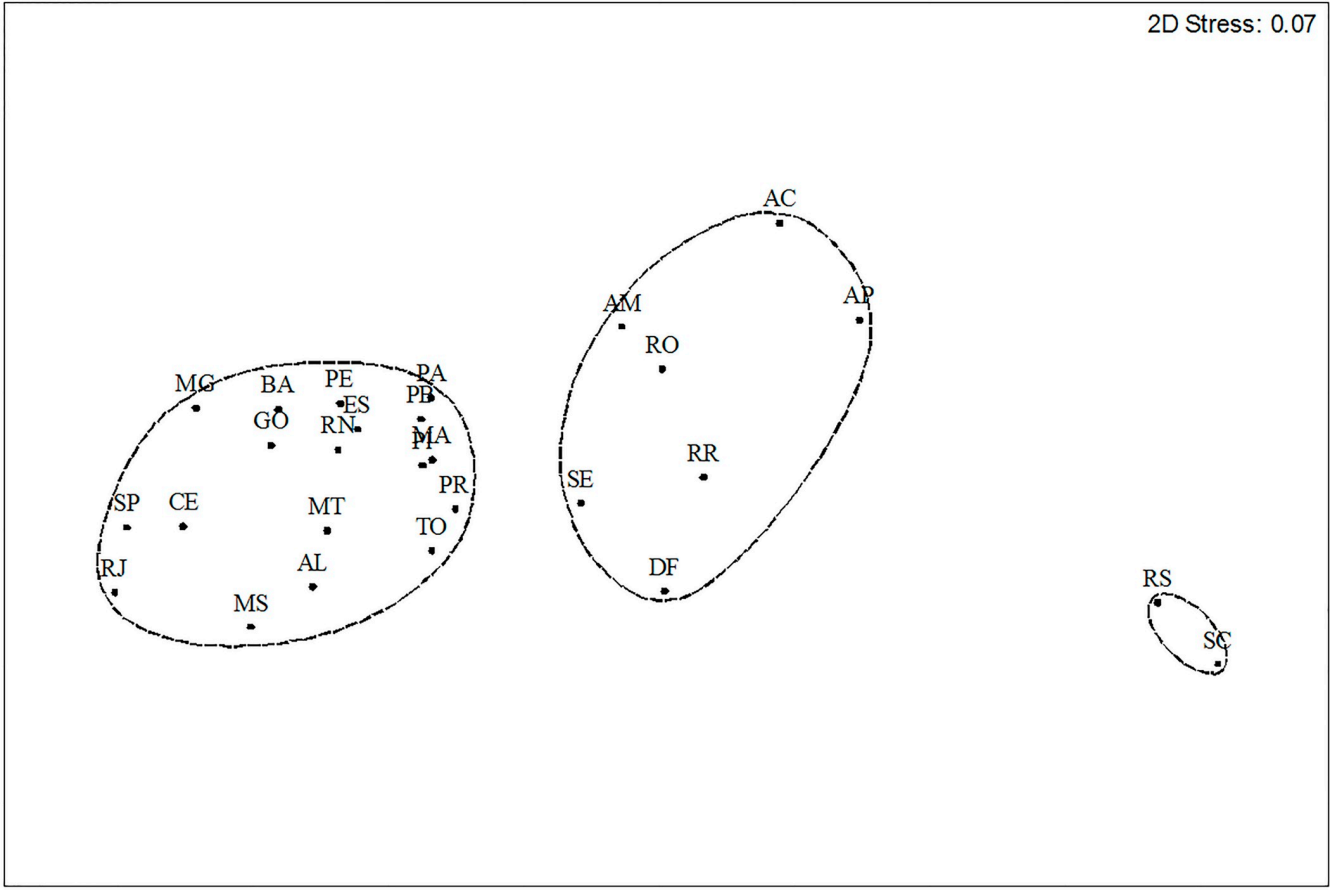
Temporal distribution and frequency of dengue reports in the states and federal district of Brazil, 1990–2017. *Abbreviations correspond to the Brazilian states and Federal District. AC—Acre; AL—Alagoas; AP—Amapá; AM—Amazonas; BA—Bahia; CE—Ceará; DF—Distrito Federal; ES—Espírito Santo; GO—Goiás; MA—Maranhão; MT—Mato Grosso; MS—Mato Grosso do Sul; MG—Minas Gerais; PA—Pará; PB—Paraíba; PR—Paraná; PE—Pernambuco; PI—Piauí; RR—Roraima; RO—Rondônia; RJ—Rio de Janeiro; RN—Rio Grande do Norte; RS—Rio Grande do Sul; SC—Santa Catarina; SP—São Paulo; SE—Sergipe; TO–Tocantins.

With a cutoff at 85% similarity between the frequency of notifications and the annual distribution of notifications in the period, three clusters were obtained. More prominently, the states of Santa Catarina and Rio Grande do Sul (Southern Region) differed from the other states. A second group gathered the states of Acre, Amazonas, Amapá, Rondônia, Roraima, Sergipe and the Federal District. The frequency of dengue in the other states was similar (Fig 5).

## Discussion

Dengue has become one of the greatest public health challenges in Brazil in the last two decades. The epidemiology of the disease presented important changes, with the highest number of cases and hospitalizations, with epidemics of great magnitude, the worsening of the process of dissemination of the transmission and the occurrence of severe cases affecting people at extreme ages (children and the elderly) [5, 24, 25]. Epidemics have shown a cyclical pattern in the occurrence from 1990 to 2017, with interepidemic periods of 3 or 4 years that have decreased over the years. The epidemic peak of 2015 illustrates the characteristics of dengue occurrence in the country in the last decade, with the trend of increasingly intense epidemic peaks, parallel to the shortening of the interepidemic periods. However, analysis by regions of the country shows that these intervals differ from region to region. A recent study carried out in Fortaleza (state of Ceará) addresses the cyclical, seasonal nature (peaks during the summer) and is dependent on the context of dengue transmission [26].

In the period between 1990 and 2016, there was an increase in the incidence and consequent increase of dengue burden and the increase in the Disability-adjusted life-years (DALYs) of Neglected Tropical Diseases (NTDs).Dengue represented the third highest proportion of DALY in the period and the main cause among NTDs in 2016, with the highest absolute number of new cases in Brazil [27]. DALY increased more than double in all Brazilian states in the period from 2000 to 2015 [28]. The dengue lethality rate was relatively low [25, 28]. It should be borne in mind that dengue contributes to the loss of healthy life years due to its high incidence, affects all age groups and causes disability during symptomatic infection [28].

Almost half of the reports of dengue (49%) of the period evaluated were recorded in the Southeast region. This Brazilian region has the highest population density in the country (86.92 inhabitants/km^2^), composed of the states of Espírito Santo, Minas Gerais, Rio de Janeiro and São Paulo, the latter being the most populous state in Brazil [21]. However, the highest rate (444.8 cases/100 thousand inhabitants) was observed in the Center-West region. The region with the lowest number of notifications and the lowest incidence was the Southern region. The states of Santa Catarina and Rio Grande do Sul (Fig 4) differed from the other states. The South region differs from the other parts of the national territory, considering the better urbanization, sanitation and the temperate mesothermal climate, with a winter season marked by low temperatures, and frequent days with minimum near 0°C [21], which impacts on the population of the vector and breaks viral transmission.

Similar results on the occurrence of dengue in the Brazilian regions and the positive relationship with population density and climate were reported by Dias [29]. The Brazilian geographic regions present seasonality associated with the incidence of dengue, with peaks generally observed during the warmer and humid months [30, 31]. These findings demonstrate how dengue is widespread in the tropics and subtropics, with risk factors influenced by local spatial variations in rainfall, temperature, humidity, and urbanization [15]. Even with fewer cases recorded in the states of Santa Catarina and Rio Grande do Sul (temperate climate), attention should be paid to climate change and to dengue cases in these regions. However, attention should be given to this region not only for climate conditions but could serve to identify potentialities and potential weaknesses of the National Dengue Control Program (*Programa Nacional de Controle da Dengue*, *PNCD*). Evidence has pointed to an increasing epidemic potential of dengue in temperate regions, influenced by the increase of diurnal temperature range (DTR) [32]. It is important to know the climatic information to predict possible epidemic periods [33].

The dynamics of viral circulation in the country was characterized by simultaneous circulation and with alternation in the predominance of viral serotypes in the epidemic years. In 1998, there was a predominance of the DENV-2 serotype, introduced in Brazil in 1990, when the first cases of Dengue Hemorrhagic Fever were confirmed [34].

The 2002 epidemic reflected the introduction of the DENV-3 serotype in the country. The introduction and predominance of DENV-3 marks the increase in the occurrence of severe forms in the disease. The 2008 epidemic was associated with recirculation and predominance of DENV-2, and the main change observed in the epidemiology of the disease during this period was the increase in severe cases in children. In the 2010 epidemic, the DENV-1 serotype predominated and the main change in epidemiology was the increase in the mortality rate of the disease [35]. From this year the autochthonous transmission of serotype DEN-4 began in the country.

An investigation of the spatial and temporal dispersion of DENV-1, 2 and 3 serotypes indicated introductions and co-circulation of different serotypes and genotypes at intervals of 7 to 10 years [36]. This dynamic of viral dispersion was determined by air transport of humans and/or mosquito vectors and eggs, important factors for the occurrence of dengue.

The deforestation rate may be associated with the incidence of vector-borne diseases. Our results suggest that the northern area of Brazil (approximately equivalent to the Amazon region) is more likely to receive and subsequently export the dengue virus to other regions.

Regarding the dengue prevention and control programs in Brazil, the Ministry of Health developed and implemented the *PNCD*. This has been conceived for understanding that dengue is not a disease that can be eradicated in the short term, pointing to the objectives of reducing *A*. *aegypti* infestation, reducing the incidence of dengue and reducing lethality due to dengue hemorrhagic fever [37], to minimize impact of disease.

Despite the efforts and intensification of the control measures, there are critiques on the effectiveness of the *PNCD* [38] based on the increase in the last years of the number of severe cases, hospitalizations and deaths [27, 28, 35, 39]. The incidence and mortality indices point to the need to improve the organization of response to dengue epidemics in an attempt to avoid severe cases, requiring an effort to mobilize managers and the population, with a permanent training program for health professionals, as well as intensification of intersectoral prevention and incentive actions for popular participation.

The increase in the incidence of dengue and the manifestation of epidemics devastating small and large urban centers, bring insecurity to the Brazilian population. In this context, Teixeira [40] emphasizes that the demand for medical care goes beyond the capacity of services. The quality of care for affected patients is lower than desired and there is distrust of the population in the leaders and managers of the health services, which makes it even more difficult to manage during periods of crisis, thus establishing a framework of fragility of the public health.

To reverse the trend of dengue transmission expansion and the endemic circulation of the four virus serotypes, new strategies are needed. To be effective they must be based on reliable information on dengue. In order to make this information more precise, the WHO published in December 2018 “*A Toolkit for national dengue burden estimation*”, a tool to standardize and guide countries to better estimate their burden of dengue by combining existing data from surveillance systems with research in progress [41].

Effective and sustainable vector control is the current challenge to reduce the burden of dengue in the population. In order to achieve this, integrated and alternative vector control strategies are needed [42–44]. The importance of intersectoral actions is reiterated and popular participation is encouraged. It is from this perspective that the global strategy of the World Health Organization (WHO) for dengue prevention aims to reduce mortality rates by 50% by 2020. These measures will be carried out, in particular, through early diagnosis and appropriate management of severe cases. Measures should also be taken to reduce morbidity by 25% by better outbreak prediction and detection through integrated management of epidemiological and entomological surveillance of vector control and combat [45]. Using the integrated vector management approach using community-based methods and adapted to the local context is one of the strategies recommended to achieve these goals. In order to boost vector control and combat capacity worldwide, WHO has developed the Global Vector Control Response 2017–2030 [46]. This plan provides guidance and strategies to countries to strengthen vector control, focused on four pillars: (1) integrated vector and disease surveillance, (2) vector control, (3) community engagement and mobilization, and (4) inter- and intra-sectoral collaboration.

The weaknesses/limitations of this study are related to epidemiological studies using secondary information sources. The possibility of underreporting of dengue cases cannot be ruled out. National surveillance systems may not capture all dengue infections, for epidemiological reasons or for sociodemographic factors. For example, individuals who are asymptomatic or have mild symptoms that do not seek care or treatment. Also, symptomatic individuals who choose to self-medicate or seek treatment at a health facility and who are misdiagnosed as another febrile illness. Another factor is incorrect records, with incomplete data and lack of reporting by private clinics [41].

Given that global, regional, national, and local data for population health indicators are needed to monitor health and guide resource allocation [47], the results presented in this study can guide and contribute to decisions on health policies, in monitoring the effectiveness and impact of health interventions directed at dengue prevention and vector control actions.

## References

[1] Bhatt S, Gething PW, Brady OJ, Messina JP, Farlow AW, Moyes CL, et al The global distribution and burden of dengue. Nature 2013; 496:504–507. 10.1038/nature12060 23563266 PMC3651993

[2] Hotez PJ, Alvarado M, Basáñez MG, Bolliger I, Bourne R, Boussinesq M, et al The global burden of disease study 2010: interpretation and implications for the neglected tropical diseases. PLoS Negl Trop Dis 2014; 8(7):e2865 10.1371/journal.pntd.0002865 25058013 PMC4109880

[3] World Health Organization. Dengue and Severe Dengue [Internet]. World Health Organization; 2018a. [cited 20 Nov 2018]. Available from: https://www.who.int/en/news-room/fact-sheets/detail/dengue-and-severe-dengue

[4] Brady OJ, Gething PW, Bhatt S, Messina JP, Brownstein JS, Hoen AG, et al Refining the global spatial limits of dengue virus transmission by evidence-based consensus. PLoS Negl Trop Dis 2012; 6(8):e1760 10.1371/journal.pntd.0001760 22880140 PMC3413714

[5] Brazilian Ministry of Health. Dengue [Internet]. Brasília: Brazilian Ministry of Health; 2018 [cited 17 Apr 2018]. Available from: http://portalms.saude.gov.br/saude-de-a-z/dengue

[6] Brazilian Ministry of Health. Guia de Vigilância em Saúde: volume único [Internet]. 2ª ed Brasília: Brazilian Ministry of Health; 2017a 705 p. [cited 28 Jun 2018]. Available from: http://portalarquivos.saude.gov.br/images/pdf/2017/outubro/06/Volume-Unico-2017.pdf

[7] Guzman MG, Harris E. Dengue. The Lancet 2015; 385(9966):453–465. 10.1016/S0140-6736(14)60572-9 25230594

[8] Shepard DS, Undurraga EA, Halasa YA, Stanaway JD. The global economic burden of dengue: a systematic analysis. Lancet Infect Dis 2016; 16(8):935–941. 10.1016/S1473-3099(16)00146-8 27091092

[9] Brazilian Ministry of Health. Saúde Brasil 2015/2016: uma análise da situação de saúde e da epidemia pelo vírus Zika e por outras doenças transmitidas pelo Aedes aegypti [Internet]. Brasília: Brazilian Ministry of Health; 2017b 387 p. [cited 17 Jun 2018]. Available from: http://portalarquivos2.saude.gov.br/images/pdf/2017/maio/12/2017-0135-vers-eletronica-final.pdf

[10] Zubieta-Zavala A, López-Cervantes M, Salinas-Escudero G, Ramírez-Chávez A, Castañeda JR, Hernández-Gaytán SI, et al Economic impact of dengue in Mexico considering reported cases for 2012 to 2016. PLoS Negl Trop Dis 2018; 12(12):e0006938 10.1371/journal.pntd.0006938 30550569 PMC6310288

[11] Bangert M, Latheef AT, Dev Pant S, Nishan Ahmed I, Saleem S, Nazla Rafeeq F, et al Economic analysis of dengue prevention and case management in the Maldives. PLoS Negl Trop Dis 2018;12(9):e0006796 10.1371/journal.pntd.0006796 30260952 PMC6177194

[12] Gubler DJ. Dengue, urbanization and globalization: the unholy trinity of the 21st century. Trop Med Health 2011; 39(Supl 4):S3–S11. 10.2149/tmh.2011-S05 22500131 PMC3317603

[13] Messina JP, Brady OJ, Pigott DM, Golding N, Kraemer MUG, Scott TW, et al The many projected futures of dengue. Nat Rev Microbiol 2015; 13(4):230–239. 10.1038/nrmicro3430 25730702

[14] Wilder-Smith A, Gubler DJ. Geographic expansion of dengue: the impact of international travel. Med Clin North Am 2008; 92(6):1377–1390 10.1016/j.mcna.2008.07.002 19061757

[15] World Health Organization. Dengue Control [Internet]. World Health Organization; 2018b. [cited 13 Oct 2018]. Available from: http://www.who.int/denguecontrol/en/

[16] Brazilian Ministry of Health. Dengue: diagnóstico e manejo clínico: adulto e criança [Internet]. 5ª ed Brasília: Brazilian Ministry of Health; 2016 58 p. [cited 09 Jun 2018]. Available from: http://portalarquivos2.saude.gov.br/images/pdf/2016/janeiro/14/dengue-manejo-adulto-crianca-5d.pdf

[17] Antonio FJ, Itami AS, Picoli S, Teixeira J, Mendes R. Spatial patterns of dengue cases in Brazil. PloS ONE 2017; 12(7):e0180715 10.1371/journal.pone.0180715 28715435 PMC5513438

[18] Lopes N, Nozawa C, Linhares REC. Características gerais e epidemiologia dos arbovírus emergentes no Brasil. Rev Pan-Amaz Saude 2014; 5(3):55–64. 10.5123/S2176-62232014000300007

[19] Deininger LSC, Lucena KDT, Figueiredo DCMM, Silva CC, Oliveira AEC, Anjos UU. A sala de situação da dengue como ferramenta de gestão em saúde. Saúde debate 2014; 38(100):50–56. 10.5935/0103-104.20140013

[20] Instituto Brasileiro de Geografia e Estatistica. Estimativas de população [internet]. Brazil. [cited 06 Mar 2019]. Available from: https://www.ibge.gov.br/

[21] Instituto Brasileiro de Geografia e Estatistica. Mapas IBGE [internet]. Brazil. [cited 12 Nov 2018]. Available from: https://portaldemapas.ibge.gov.br

[22] Clarke KR, Gorley RN. Primer: getting started with v6. Plymouth routines in multivariate ecological research–PRIMER-E Ltd; 2005.

[23] Hammer O, Harper DAT, Rian PD. Past: palaeonthological statistics software package for education and data analysis. Palaeontologia Electronica, vol. 4, issue 1, art. 4: 9pp., 178kb Version. 1.37; 2001 [cited 11 Nov 2018]. Available from: https://palaeo-electronica.org/2001_1/past/past.pdf

[24] Teixeira MG, Siqueira JB Jr, Ferreira GLC, Bricks L, Joint G. Epidemiological Trends of Dengue Disease in Brazil (2000–2010): A Systematic Literature Search and Analysis. PLoS Negl Trop Dis 2013; 7(12):e2520 10.1371/journal.pntd.0002520 24386496 PMC3871634

[25] Paixão ES, Costa MCN, Rodrigues LC, Rasella D, Cardim LL, Brasileiro AC et al Trends and factors associated with dengue mortality and fatality in Brazil. Rev Soc Bras Med Trop 2015; 48(4):399–405. 10.1590/0037-8682-0145-2015 26312928

[26] MacCormack-Gelles B, Lima Neto AS, Sousa GS, Nascimento OJ, Machado MMT, Wilson ME, et al Epidemiological characteristics and determinants of dengue transmission during epidemic and non-epidemic years in Fortaleza, Brazil: 2011–2015. PLoS Negl Trop Dis 2018; 12(12):e0006990 10.1371/journal.pntd.0006990 30507968 PMC6292645

[27] Martins-Melo FR, Carneiro M, Ramos AN Jr., Heukelbach J, Ribeiro ALP, Werneck GL. The burden of Neglected Tropical Diseases in Brazil, 1990–2016: A subnational analysis from the Global Burden of Disease Study 2016. PLoS Negl Trop Dis 2018; 12(6):e0006559 10.1371/journal.pntd.0006559 29864133 PMC6013251

[28] Araújo VEMD, Bezerra JMT, Amâncio FF, Passos VMDA, Carneiro M. Increase in the burden of dengue in Brazil and federated units, 2000 and 2015: analysis of the Global Burden of Disease Study 2015. Rev Bras Epidemiol 2017; 20(Supl 1):205–216. 10.1590/1980-5497201700050017 28658384

[29] Dias JP. Avaliação da efetividade do Programa de Erradicação do Aedes aegypti. Brasil, 1996–2002 [doctoral thesis] [Internet]. Salvador: Instituto de Saúde Coletiva da Universidade Federal da Bahia; 2006 [cited 20 Sep 2018]. Available from: https://repositorio.ufba.br/ri/bitstream/ri/10392/2/Tese_final_03_03_06-sec.pdf

[30] Johansson MA, Dominici F, Glass GE. Local and global effects of climate on dengue transmission in Puerto Rico. PLoS Negl Trop Dis 2009; 3(2):e382 Epub 2009/02/18. 10.1371/journal.pntd.0000382 19221592 PMC2637540

[31] Descloux E, Mangeas M, Menkes CE, Lengaigne M, Leroy A, Tehei T, et al Climate-based models for understanding and forecasting dengue epidemics. PLoS Negl Trop Dis 2012; 6(2):e1470 Epub 2012/02/22. 10.1371/journal.pntd.0001470 22348154 PMC3279338

[32] Liu-Helmersson J, Stenlund H, Wilder-Smith A, Rocklöv J. Vectorial Capacity of Aedes aegypti: Effects of Temperature and Implications for Global Dengue Epidemic Potential. PLoS ONE 2014; 9(3):e89783 10.1371/journal.pone.0089783 24603439 PMC3946027

[33] Lowe R, Stewart-Ibarra AM, Petrova D, García-Díez M, Borbor-Cordova MJ, Mejía R, et al Climate services for health: predicting the evolution of the 2016 dengue season in Machala, Ecuador. The Lancet Planetary Health 2017; 1:e142–e151. 10.1016/S2542-5196(17)30064-5 29851600

[34] Siqueira JB, Martelli CM, Coelho GE, Simplício AC, Hatch DL. Dengue and Dengue Hemorrhagic Fever, Brazil, 1981–2002. Emerg Infect Dis. 2005; 11(1):48–53. 10.3201/eid1101.031091 15705322 PMC3294356

[35] Siqueira JB, Vinhal LC, Said RFC, Hoffmann JL, Martins J, Barbiratto SB et al. Dengue no Brasil: tendências e mudanças na epidemiologia, com ênfase nas epidemias de 2008 e 2010. In: Brasil. Ministério da Saúde. Saúde Brasil 2010: uma análise da situação de saúde e de evidências selecionadas de impacto de ações de vigilância em saúde [Internet]. Brasília: Ministério da Saúde; 2011. p. 157–171. [cited 20 Aug 2018]. Available from: http://bvsms.saude.gov.br/bvs/publicacoes/saudebrasil2010.pdf

[36] Nunes MRT, Palacios G, Faria NR, Sousa EC Jr, Pantoja JA, Rodrigues SG et al Air Travel Is Associated with Intracontinental Spread of Dengue Virus Serotypes 1–3 in Brazil. PLoS Negl Trop Dis 2014; 8(4):e2769 10.1371/journal.pntd.0002769 24743730 PMC3990485

[37] Brazilian Ministry of Health. Fundação Nacional de Saúde. Programa Nacional de Controle da Dengue–PNCD [Internet]. Brasília: Brazilian Ministry of Health/Funasa; 2002 34 p. [cited 14 Jul 2018]. Available from: http://bvsms.saude.gov.br/bvs/publicacoes/pncd_2002.pdf

[38] Barreto ML, Teixeira MG, Bastos FI, Ximenes RA, Barata RB, Rodrigues LC. Successes and failures in the control of infectious diseases in Brazil: social and environmental context, policies, interventions, and research needs. The lancet 2011; 377(9780):1877–1889. 10.1016/S0140-6736(11)60202-X 21561657

[39] Moraes GH, Fátima Duarte E, Duarte EC. Determinants of mortality from severe dengue in Brazil: a population-based case-control study. Am J Trop Med Hyg 2013; 88(4):670–676. 10.4269/ajtmh.11-0774 23400577 PMC3617850

[40] Teixeira MG. Few characteristics of dengue's fever epidemiology in Brazil. Rev Inst Med trop S. Paulo 2012; 54(Supl 18):1–4. 10.1590/S0036-46652012000700002 23011449

[41] World Health Organization. A Toolkit for national dengue burden estimationum [Internet]. Geneva: World Health Organization; 2018c [cited 02 Dec 2018]. Available from: http://apps.who.int/iris/bitstream/handle/10665/277257/WHO-CDS-NTD-VEM-2018.05-eng.pdf?ua=1

[42] Achee NL, Gould F, Perkins TA, Reiner RC Jr., Morrison AC, Ritchie SA, et al A Critical Assessment of Vector Control for Dengue Prevention. PLoS Negl Trop Dis 2015; 9(5):e0003655 10.1371/journal.pntd.0003655 25951103 PMC4423954

[43] Roiz D, Wilson AL, Scott TW, Fonseca DM, Jourdain F, Müller P, et al Integrated Aedes management for the control of Aedes-borne diseases. PLoS Negl Trop Dis 2018; 12(12):e0006845 10.1371/journal.pntd.0006845 30521524 PMC6283470

[44] Achee NL, Grieco JP, Vatandoost H, Seixas G, Pinto J, Ching-NG L, et al Alternative strategies for mosquito-borne arbovírus control. PLoS Negl Trop Dis 2019; 13(1):e0006822 10.1371/journal.pntd.0006822 30605475 PMC6317787

[45] World Health Organization. Global strategy for dengue prevention and control 2012–2020 [internet]. Geneva: World Health Organization; 2012 [cited 15 Dec 2018]. Available from: http://apps.who.int/iris/bitstream/handle/10665/75303/9789241504034eng.pdf?sequence=1

[46] World Health Organization. Global vector control response 2017–2030 [internet]. Geneva: World Health Organization; 2017 [cited 20 Dec 2018]. Available from: http://apps.who.int/iris/bitstream/handle/10665/259205/9789241512978-eng.pdf?sequence=1

[47] Stevens GA, Alkema L, Black RE, Boerma JT, Collins GS, Ezzati M, et al Guidelines for Accurate and Transparent Health Estimates Reporting: the GATHER statement. PLoS Med 2016; 13(6):e1002056 10.1371/journal.pmed.1002056 27351744 PMC4924581

