# Supplementary material for: Spatial and temporal distribution of dengue in Brazil, 1990 - 2017
Source: PLoS One. 2020 Feb 13;15(2):e0228346. doi: 10.1371/journal.pone.0228346 (PMC7018131; doi:10.1371/journal.pone.0228346)
Supplement: Attachment — Submitted filename: Letter_responses to the review comments.docx [file pone.0228346.s001.docx]

**RESPONSES TO REFEREES’ COMMENTS AND ALTERATIONS MADE IN THE MANUSCRIPT**

**Methods**

**Reviewer #1:** The methods section can be much shorter, and presenting only the actual methods of the analysis. Context, figure 1 and figure 2 are interesting, but not so much for a scientific presentation

**Answer:** The methods section was shortened. Figure 1 and Figure 2 were deleted.

**Reviewer #3:** The manuscript consolidates information from the Brazilian Ministry of Health on the incidence of dengue in the country, by state, over a period of almost 30 years, from 1990 to 2017. It is a useful work because it allows to draw a historical panorama of the dynamics of this arbovirose in the country, unfortunately a protagonist when this arbovirus is taken into account. Evaluation of this historical scenario included the identification of transmission pattern and of changes in this pattern over time. The other positive aspect of the work was to use and make available official documents on the subject. However, there are some issues that merit treatment with some additional detail, such as the deepening in the discussion of regional differences and a better conceptualization of the term 'internalization'. Another point that should be addressed in more detail is the discussion about the existence or not of reduction of interepidemic periods. These and other particular aspects are commented below, in order of appearance in the text.

lines 31-33 (Abstract), “Dengue incidence increased in all Brazilian regions and epidemics showed a cyclical pattern, with intervals of 3 to 4 years...” – See comment on lines 214-216, which address the need to observe intervals by region.

**Answer**: Text was changed as suggested.

lines 53-54 (Author Summary), “However, actions that minimize the suffering and impacts caused by dengue need to be improved and continued.” - This is more of a wish of the authors than the motivation, or conclusion, of the study. By the way, it is worth highlighting an aspect that is clear to managers, at least theoretically: that dengue transcends the health area and depends on intersectoral actions to be controlled. This item could also mention the other emerging arboviruses, being currently transmitted by Aedes aegypti in the country, and causing epidemics.

**Answer**: Text was changed as suggested.

lines 71-73, “The vector is cosmopolitan and occurs in tropical and subtropical countries where climatic and environmental conditions favor its dispersal and development.” - The climate and the environmental conditions are not the only relevant factors that favor the dispersion of the vector. It is important to mention aspects related to the disorganized growth of cities, which greatly contribute to the vector dissemination (irregular water supply, precarious sanitation, deficiencies in waste collection, unfinished and abandoned constructions etc).

**Answer**: Text change as suggested. We mentioned other aspects that contribute to the spread of the vector.

lines 101-103, “The investigation of the epidemiological profile of dengue is important so that its magnitude is known, for the definition of public policies and the timely allocation of resources in the actions of control and prevention.” - I'm not convinced that this is the best argument for the work done. Detailed monitoring is already done routinely in the country, and the proof is the fact that this manuscript is using MoH data. It may be worth adding the need (or opportunity) to make a more general and comprehensive assessment of the data that is generated in routine epidemiological surveillance in the country.

**Answer**: Text was changed as suggested.

line 187, but also lines 75 and 211, ‘interiorization’– It would be correct to speak of 'spread', or 'dissemination' of dengue accross the country. However, I have doubts about the term 'internalization'. This term, 'internalization', is being used here with the meaning of dissemination to non-coastal states. But there are 'internal' states that have been affected since the beginning of the assessed period. Looking at the maps, what seems most evident is the growing involvement of the Central-West region (beyond dengue general dissemination, with increasing numbers of cases over time). In addition, in general the term 'internalization' is applied to another aspect of dengue in Brazil, which is the spread to smaller cities, away from the main metropolises of the country. Therefore, I suggest checking the term and informing the concept used at the beginning of the text, as a clarification.

**Answer**: Text was changed as suggested. We replaced the term 'interiorization' with 'spread' and 'dissemination'.

Figure 6, this figure is very elucidative. Two suggestions: (a) to increase the size of the numbers indicating the years and (b) to place on top, in larger size, the maps corresponding to the first and to the last evaluation years, 1990 and 2017 (I did not understand the rationale of including at the top the maps corresponding to 1990 and 1991).

**Answer**: We made suggested changes. The numbers indicating the years were enlarged and a map was presented in larger size to help identify states and regions of the country.

lines 214 – 216, “The epidemic peak of 2015 illustrates the characteristics of dengue occurrence in the country in the last decade, with the trend of increasingly intense epidemic peaks, parallel to the shortening of the interepidemic periods.” - I think this aspect should be analyzed in greater detail, by region. Figure 4 shows the incidence of dengue by region, but there is no figure showing the number of cases per region. The issue is that for a long time the most affected region was the more populous Southeast region, and the dynamics of dengue in the Southeast has a very large influence on the national dynamics. Then, if we look closely at Figure 4, we will see that the interepidemic periods are distinct in the different regions. Although they have narrowed in the Southeast region, in several other regions of the country the interval of 3-4 years seems to remain. Perhaps the graphical presentation by region compared to the national profile helps the visualization.

**Answer**: Changes were made to Figure 4 and the text as suggested. A graphical presentation of the reported cases and dengue incidence by region was made, compared with the national profile. Epidemic periods were analyzed in more detail by region.

lines 233 – 235, “The South region differs from the other parts of the national territory, because it presents a temperate mesothermal climate...” - The higher purchasing power of these states, which is reflected in better urbanization and sanitation conditions, for example, should also contribute to the distinct dynamics of dengue in the Southern Region. It is worth remembering that Aedes aegypti is an essentially domestic mosquito.

**Answer**: Text was changed as suggested. Urbanization and sanitation conditions were added as factors that contribute to the distinct dengue dynamics in the Southern region.

lines 243 – 245, “Even with fewer cases recorded in the states of Santa Catarina and Rio Grande do Sul (temperate climate), attention should be paid to climate change and to dengue cases in these regions.” - Attention should rather be given to the Southern Region, but not only because of the climate. It would serve to identify errors and successes of the national control program, now well structured (and only now being applied in the South region, which began to be affected, in fact, recently). It would also serve to dissociate, at least partially, the climatic component of economic and social issues, because in general they are states with a better urban structuring.

**Answer**: Text was changed as suggested. Attention to the southern region should be given not only because of the climate.

lines 256-257, “...and the main change observed in the epidemiology of the disease during this period was the increase in severe cases in children.” - Just a comment: probably due to the exhaustion of susceptibles in the older groups.

**Answer**: No change.

lines 263 – 264, “This dynamics of viral dispersion was determined by air transport of humans and/or mosquito vectors, important factors for the occurrence of dengue.” The importance of the passive dissemination of *Aedes aegypti* by means of dry eggs transportation is well recognized.

**Answer**: Changes in the text. The transport of dry eggs is also responsible for the passive spread of Aedes aegypti.

lines 266-267, “...the northern area of Brazil ... is more likely to receive and subsequently export the virus to other regions.” - I did not understand. Does the text mean the dengue virus, or other arboviruses?

**Answer**: Changes in the text. The dengue virus.

lines 287-288, “This [PNCD] has been conceived from a permanent program perspective for understanding that dengue is not a disease that can be eradicated in the short term, pointing...” - In fact PNCD was the plan designed after the realization that it would not be possible to eradicate again Aedes aegypti from the country. In summary, PNCD objectives were to keep the vector under control and to minimize the impacts of the disease.

**Answer**: Text was changed as suggested.

lines 302-305, “The incidence and mortality indices point to the need to improve the organization of response to dengue epidemics in an attempt to avoid severe cases, requiring an effort to mobilize managers and the population, with a permanent  training program for health professionals” – In relation to “response to dengue epidemics” – this sentence shows part of the solution. The other component would be the investment in prevention actions and the encouragement of popular participation - independently of epidemics. There is yet another aspect, which is the intersectoral action.

**Answer**: Text change as suggested. The intensification of intersectoral prevention actions and the encouragement of popular participation are important aspects to prevent the occurrence of epidemics and the response to them.

lines 319-321, “Effective and sustainable vector control is the current challenge to reduce the burden of dengue in the population. In order to achieve this, integrated and alternative vector control strategies are needed [44 - 46].” - It is worth commenting on sustainable strategies, which include popular participation and are not limited to technical solutions, but also work on prevention. The intersectoriality of the problem is recognized, at least theoretically, by the control programs. Therefore, it should, in fact, be accompanied by the intersectoriality of control actions.

**Answer**: Text change as suggested. We reinforce the importance of intersectoral actions and the encouragement of popular participation to reduce the burden of dengue in the population.

**Minor comments**

lines 80-81, “It is observed a growing incidence and geographic expansion of outbreaks and epidemics associated with the lack of effective vector control...” could be ‘A growing incidence and geographic expansion of dengue outbreaks and epidemics is associated with the lack of effective vector control...’

**Answer**: Text was changed as suggested.

line 88, “urbanization” - could be 'disorganized urbanization'

**Answer**: Replaced 'urbanization' with 'disorganized urbanization'.

line 91, “Chikungunya” is ‘chikungunya’ (only Zika should be written in upper case, since this name refers to a place, the Zika forest, at Uganda)

**Answer**: Done.

line 97, reference 18 - Reference 18 is not the best to present mayaro circulation in Brazil, since it deals with experimentation with the virus and the Wolbachia.

**Answer**: Reference 18 was replaced by another that shows the circulation of mayaro in Brazil.

Figures 1 and 2 can be simplified and presented in a single figure.

**Answer**: As suggested, Figures 1 and 2 were deleted.

line 221, “...Neglected Tropical Diseases (NTDs), representing the...” – (suggestion: to split the sentence) "... Neglected Tropical Diseases (NTDs). Dengue represented the third..."

**Answer**: Text was changed as suggested.

line 230, “...São Paulo, the latter being the most populous states in Brazil” – should be ‘...São Paulo, the latter being the most populous state in Brazil...’

**Answer**: Text was changed as suggested.

**Results**

**Reviewer #1**: Please expand on the results section, this is the key part of your study. Some more data on case fatalities would be very interesting, since this is the area, where dengue can be controlled.

**Answer**: The results section was expanded. Data on dengue deaths were added.

**Reviewer #2**: (No Response).

**Reviewer #3**: see above

**Conclusions**

**Reviewer #1**: In the discussion, although very good, the different dengue control plans in Brazil are correlated with the epidemiology of dengue. This is another study, actually, and not so much a discussion. The discussion could be shortened in this section, and key conclusion points could be highlighted.

**Answer**: The text was shortened as suggested. We agree that the discussion of how dengue control plans in Brazil are correlated with epidemiology would be another study.

**Reviewer #2**: (No Response).

**Reviewer #3**: see above

**Editorial and Data Presentation Modifications**

**Reviewer #1:** Figures 1 and 2 are really not necessary for the global audience
Figures 3 and 4 need a legend and also information of the data sources

**Answer**: Figures 1 and 2 were excluded. The other figures were renumbered in the order in which they appear in the text. Subtitle and source of data were added as needed.

**Reviewer #2:** (No Response).

**Reviewer #3**: see above

**Summary and General Comments**

**Reviewer #2:** I have submitted my review as an attachment. The article has to undergo few corrections. I have mentioned them in my review.

**Response to comments from Referee # 2:**

Line 80 - 82: Please rewrite these lines and make grammatical correction. (It is observed a growing incidence and geographic expansion of outbreaks and epidemics associated with the lack of effective vector control, lifestyle changes, population growth, urbanization, globalization and international human mobility).

**Answer**: Made grammatical correction.

Line 115: The study area covered Brazil, the largest country in Latin America, with a geographical area of 8,515,759,090 Km2, in South America. -Please correct the geographical area of Brazil. It is written 8515,767 km^2^ in Wikipedia. Please see more reliable source and correct accordingly.

**Answer**: The geographical area of Brazil was corrected with data from the source cited.

In table no. 1. Express the figures in decimal points rather than using comma (,) which creates confusion.

**Answer**: Replaced comma (,) with period (.).

Line 230: São Paulo, the latter being the most populous states in Brazil. -Instead of ‘States’ write ‘state’ here.

**Answer**: Done.

Line 236-237: To conclude that low temperature impacts the population of the vector and breaks viral transmission. It would be better to ‘Cite’ this fact to existing literature.

**Answer**: Text was changed as suggested by another referee. The influence of low temperatures on the vector is well documented.

Line 254-255: The introduction and predominance of DENV-3 marks the increase in the occurrence of severe forms in the disease. In this line instead of writing ‘severe forms in the disease’ write ‘severe forms of the diseases’.

**Answer**: Done.

Line 259: Please cite the statement – From this year the autochthonous transmission of serotype DEN-4 began in the country.

**Answer**: Done. The discussion of the paragraph refers to reference number ‘35’.

Line 265 – 267: Give your arguments and evidences to justify that northern area of brazil is more likely to receive and subsequently export the virus to other regions.

**Answer**: These are only hypotheses/possibilities suggested by the authors.
